# Phase 1 randomized pharmacokinetic and safety study of a 90‐day tenofovir vaginal ring in the United States

**DOI:** 10.1002/jia2.26223

**Published:** 2024-03-05

**Authors:** Albert Y. Liu, Holly Gundacker, Barbra Richardson, Beatrice A. Chen, Craig Hoesley, Ariane van der Straten, Amanda Brown, May Beamer, Jennifer Robinson, Cindy E. Jacobson, Rachel Scheckter, Katherine Bunge, Jill Schwartz, Andrea Thurman, Jeanna M. Piper, Mark A. Marzinke, the MTN‐038 Protocol Team for the Microbicide Trials Network

**Affiliations:** ^1^ Bridge HIV San Francisco Department of Public Health San Francisco California USA; ^2^ Department of Medicine University of California San Francisco San Francisco California USA; ^3^ Statistical Center for HIV/AIDS Research & Prevention Fred Hutchinson Cancer Center Seattle Washington USA; ^4^ Departments of Biostatistics and Global Health University of Washington Seattle Washington USA; ^5^ Vaccine and Infectious Disease and Public Health Sciences Divisions Fred Hutchinson Cancer Center Seattle Washington USA; ^6^ Department of Obstetrics Gynecology and Reproductive Sciences University of Pittsburgh Pittsburgh Pennsylvania USA; ^7^ Magee‐Womens Research Institute Pittsburgh Pennsylvania USA; ^8^ University of Alabama at Birmingham Birmingham Alabama USA; ^9^ ASTRA Consulting Kensington California USA; ^10^ Division of Gynecology and Obstetrics Johns Hopkins University School of Medicine Baltimore Maryland USA; ^11^ FHI 360 Durham North Carolina USA; ^12^ CONRAD Eastern Virginia Medical School Norfolk Virginia USA; ^13^ Division of AIDS National Institutes of Health Bethesda Maryland USA; ^14^ Division of Clinical Pharmacology Department of Medicine Johns Hopkins University School of Medicine Baltimore Maryland USA; ^15^ Department of Pathology Johns Hopkins University School of Medicine Baltimore Maryland USA

**Keywords:** tenofovir, vaginal ring, pharmacokinetics, safety, microbicide, pre‐exposure prophylaxis

## Abstract

**Introduction:**

Tenofovir‐based oral pre‐exposure prophylaxis is currently approved for HIV prevention; however, adherence in women has been low. A vaginal gel containing tenofovir (TFV) demonstrated partial protection to HIV but protection was not confirmed in additional studies. Vaginal rings offer user‐controlled long‐acting HIV prevention that could overcome adherence and protection challenges. TFV may also help prevent herpes simplex virus type 2 acquisition when delivered intravaginally. We evaluated the pharmacokinetics, safety, adherence and acceptability of a 90‐day TFV ring.

**Methods:**

Between January and June 2019, Microbicide Trials Network (MTN)‐038 enrolled 49 HIV‐negative participants into a phase 1, randomized (2:1) trial comparing a 90‐day ring containing 1.4 grams (g) TFV to a placebo ring. TFV concentrations were quantified in plasma, cervicovaginal fluid (CVF), rectal fluid and cervical tissue, and TFV‐diphosphate (TFV‐DP) in cervical tissue. Used rings were analysed for residual TFV. Safety was assessed by adverse events (AEs); acceptability and adherence by self‐report.

**Results:**

Mean age was 29.5; 46 identified as cisgender‐female and three gender non‐conforming. There were no differences in the proportion of participants with grade ≥2 genitourinary AEs in the TFV versus placebo arms (*p* = 0.41); no grade ≥3 AEs were reported. Geometric mean TFV concentrations increased through day 34 in CVF/rectal fluid and day 59 in plasma, but declined across compartments by day 91. Geometric mean TFV‐DP tissue concentrations exceeded the 1000 fmol/mg target through day 56, but fell to 456 fmol/mg at day 91. Among 32 rings returned at the end of the study, 13 had no or low (<0.1 g) residual TFV. Residual TFV did not differ by socio‐demographics, sexual activity, Nugent Score or vaginal microbiota. Most participants reported being fully adherent to ring use: 85% and 81% in the TFV and placebo arms, respectively (*p* = 1.00). A majority of participants reported liking the ring (median 8 on a 10‐point Likert scale) and reported a high likelihood of using the ring in the future, if effective (median 9).

**Conclusions:**

The 90‐day TFV ring was well‐tolerated, acceptable and exceeded target cervical tissue concentrations through day 56, but declined thereafter. Additional studies are needed to characterize the higher release from TFV rings in some participants and the optimal duration of use.

## 1 INTRODUCTION

Over half of the 38 million people living with human immunodeficiency virus (HIV) worldwide are women [1]. In sub‐Saharan Africa, women and girls accounted for 63% of new HIV infections in 2020 [2]. In the United States, nearly one‐fifth of new HIV diagnoses are among women, with 57% occurring among Black women [3]. Additionally, herpes simplex virus type 2 (HSV‐2) infection is more common in women [4] and is associated with increased HIV acquisition and transmission [5, 6] and serious infection in neonates [7].

Tenofovir (TFV) is currently approved for pre‐exposure prophylaxis (PrEP) in women as daily oral tenofovir disoproxil fumarate/emtricitabine (TDF/FTC). However, oral PrEP adherence has been low across a number of studies in women, limiting effectiveness [8−11]. TFV may also help prevent HSV‐2 acquisition when delivered intravaginally [12, 13] or orally [14].

Vaginal rings offer discreet, long‐acting HIV prevention that could help overcome daily adherence issues, thus providing a viable alternative to oral tablets. An extended duration ring replaced quarterly may further reduce patient and provider burden and improve adherence. The Microbicide Trials Network (MTN)‐038 study, a collaboration between the MTN and CONRAD, evaluated the pharmacokinetics (PK), safety, adherence and acceptability of CONRAD's 90‐day TFV ring compared to placebo in healthy, HIV‐uninfected US participants assigned female at birth.

## 2 METHODS

### 2.1 Study design

MTN‐038 was a phase 1 multi‐site, two‐arm, randomized trial enrolled across three sites: University of Pittsburgh, Pittsburgh, PA; San Francisco Department of Public Health, San Francisco, CA; and University of Alabama, Birmingham, AL. The final study follow‐up was completed in September 2019. Each site received local institutional review board approval.

**Figure 1:**
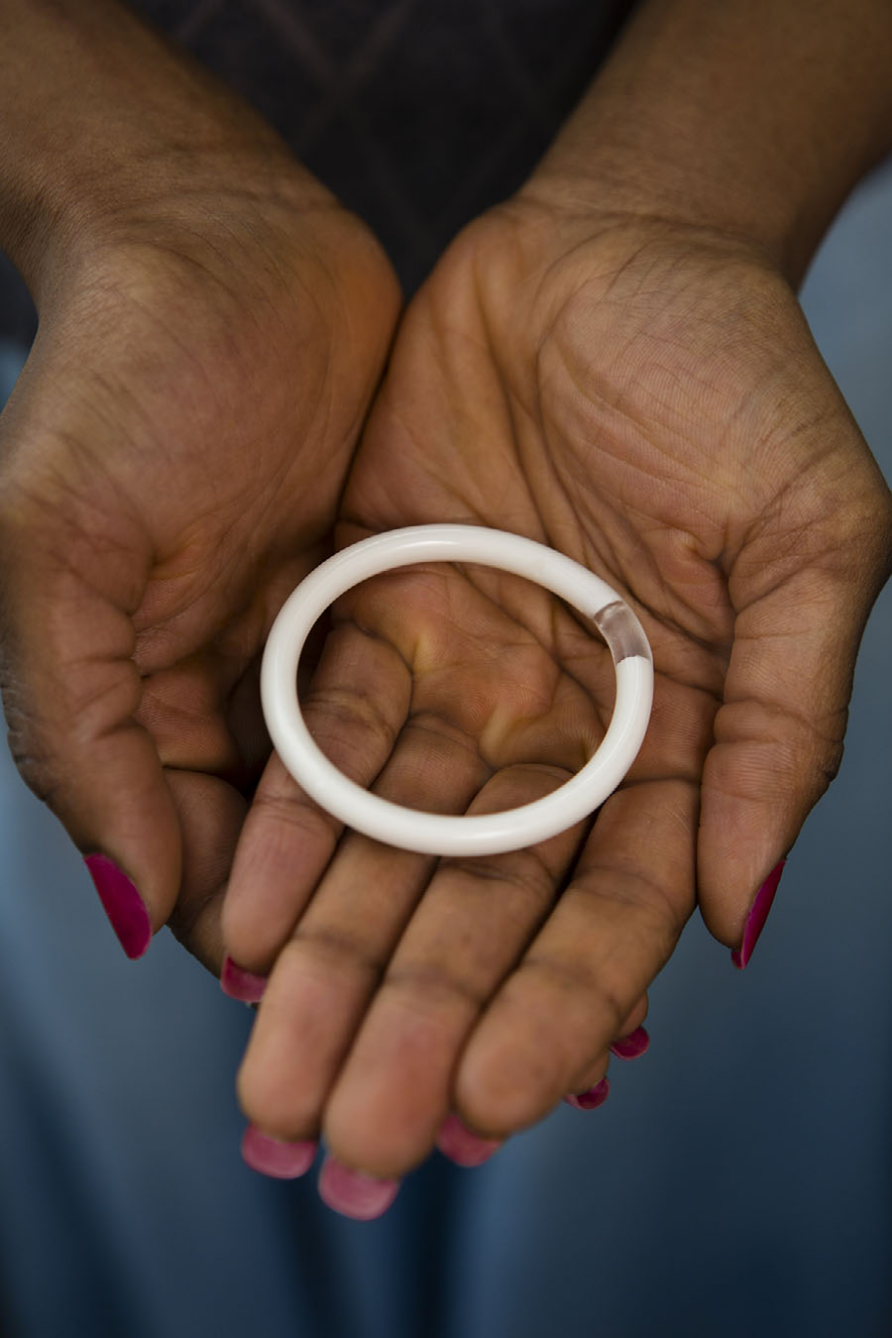
TFV vaginal ring.

TFV vaginal ring is a drug‐loaded hydrophilic polyether urethane tube with a 0.7 mm wall thickness, 5.5 mm outer cross‐sectional diameter and 55 mm outer diameter (Figure 1) [15]. This reservoir ring is loaded with 1.4 g TFV (PMPA) and uses polyurethane as a rate‐controlling membrane capable of delivering ∼10 mg/day TFV for 90 days. The placebo ring has a similar appearance, size and make‐up but does not contain active ingredients.

The primary study objectives were to evaluate safety, local and systemic PK of a TFV ring used continuously for 91 days. Primary PK endpoints included TFV concentrations in plasma, cervicovaginal fluid (CVF), rectal fluid (RF), and cervical tissue and tenofovir diphosphate (TFV‐DP) in cervical tissue. Safety evaluated the proportion of participants with grade ≥2 genitourinary adverse events (AEs) and grade ≥3 AEs, using Division of AIDS Grading Tables [16, 17]. Genitourinary AE assessments were based on self‐reported symptoms and pelvic exams. Secondary objectives evaluated adherence and acceptability of the TFV ring. An exploratory objective assessed residual TFV levels in returned rings.

Eligible participants were assigned female at birth, aged 18−45, HIV negative, using effective non‐ring‐based contraception, reported regular menses (for those not using hormonal contraception), and generally healthy. Participants were excluded if they were pregnant/breastfeeding, reported injection drug use in the past year, had used pre‐/post‐exposure HIV prophylaxis in the past 3 months, had any unresolved urinary or reproductive tract infection or any sexually transmitted infection requiring treatment, chronic/recurrent candidiasis, significant hematologic or liver function test abnormalities, creatinine clearance <60 ml/minute, positive hepatitis B surface antigen or clinically apparent grade ≥2 gynaecologic abnormalities. Participants were not required to be sexually active or have a sex partner to join, but for those who were, they had to be willing to use male condoms for penile sexual intercourse during the study.

After providing written consent and completing screening, eligible participants were randomized 2:1 to TFV or placebo ring. Study staff and participants were not informed of treatment assignments. At all visits, a clinician confirmed the correct ring placement by visualization. CVF was collected 1 and 4 hours after ring insertion, and blood, CVF and RF were obtained at a single time point for drug measurements on days 1, 7 (blood and CVF only), 14, 28 and 56. On day 91, blood, CVF and RF were collected immediately before and 4 hours after ring removal, and blood and CVF were collected at day 92. CVF and RF were clinician‐collected via a Dacron swab within 30 minutes of blood collection, and net weights were determined. Participants were randomized to cervical tissue biopsy at days 14 and 56 or days 28 and 91; net biopsy weights were recorded, and biopsies were immediately flash frozen. At day 91, used rings were cleaned and stored at −80°C until shipment for residual drug analysis. Any severe or unexpected social harms were reported to the Protocol Safety Review Team.

### 2.2 Sample and randomization

Based upon the size of similar phase 1 studies of vaginal microbicide products, we targeted 48 participants. Participants were randomized using permuted block randomization in a 2:1 ratio of active to placebo and were stratified by the site to ensure balanced product assignment.

### 2.3 Laboratory methods

TFV was quantified in plasma, CVF, RF and cervical tissue using previously described, liquid chromatographic‐tandem mass spectrometric methods by the Clinical Pharmacology Analytical Laboratory at the Johns Hopkins University School of Medicine [18−20]. Quantification of TFV‐diphosphate (TFV‐DP) in tissue was conducted using a previously described indirect enzymatic approach [21, 22]. All assays were validated in accordance with food and drug administration (FDA) bioanalytical guidelines. Lower limits of quantification (LLOQ) were as follows: plasma TFV, 0.31 ng/ml; CVF TFV, 0.625 ng/swab; RF 0.625 ng/swab; cervical tissue TFV, 0.05 ng/sample; cervical tissue TFV‐DP, 5 fmol/sample. When normalized to fluid or biopsy weights, median LLOQs were 0.008 ng/mg (interquartile range [IQR] 0.007−0.012 ng/mg) for CVF TFV; 0.035 ng/mg (IQR 0.022−0.050 ng/mg) for RF TFV; 0.004 ng/mg (IQR 0.003−0.006 ng/mg) for cervical tissue TFV and 0.392 fmol/mg (IQR 0.273−0.641 fmol/mg) for cervical tissue TFV‐DP.

Residual TFV content in returned rings was determined by Particle Sciences (Lubrizol). TFV vaginal rings were cut into 5−8 mm sections before dissolving residual TFV in 42−55 ml of 0.1 N NaOH and diluting to 100 ml with 50 mM potassium phosphate buffer, pH 6.0.  An aliquot of this solution was further diluted 50‐fold before filtration through 0.2 μm nylon membrane prior to analysis by high‐performance liquid chromatography as described previously [15, 23], with LLOQ of 1.02 μg.  In post‐hoc analysis, vaginal microbiota were evaluated using Nugent criteria and quantitative polymerase chain reaction (qPCR). Briefly, swabs were used to obtain samples from lateral vaginal wall during four visits: prior to ring insertion at enrolment, at days 28 and 56, and prior to ring removal at day 91. One Dacron swab was used to prepare a slide that was Gram‐stained and evaluated using the Nugent criteria, and one flocked swab was collected for qPCR and placed in a 2‐ml cryovial for storage at ≤−70°C [24]. The flocked swab was extracted for DNA and subjected to qPCR with specific primer sets targeting 16S ribosomal RNA genes of various microbiota and detected with SYBR green technology, as previously described [24, 25, 26, 27, 28].

### 2.4 Pharmacokinetic and statistical analysis

Participant characteristics were summarized using descriptive statistics. For the primary safety objective (all participants who inserted the ring), we compared the proportion of participants with AE endpoints in TFV versus placebo ring arms using Fisher's exact test. Plasma, CVF, RF and cervical tissue drug concentrations are summarized for participants in the TFV arm who have concentration results from at least two time points. Geometric mean concentrations for each sample type across time points were calculated, and concentration‐time curves were created with bars to indicate the exponentiated mean +/− standard deviation (SD) of log values. Area under the concentration‐time curves (AUC) in plasma, CVF and RF were calculated among participants completing the study product through day 92 (AUC_0‐92_) using the trapezoidal method. Peak concentration (C_max_) and time to peak concentration (T_max_) among participants completing up to day 91 were also determined. TFV and TFV‐DP concentrations reported as below the LLOQ were imputed with a value equivalent to half the sample‐specific LLOQ.

Participants were classified as fully adherent if they reported having kept the ring inserted at all times, without any discontinuation, hold or ring outage. Acceptability was assessed using a 10‐point Likert scale asking how much the participant liked the ring and how likely they would be to use it in the future if found to be effective for HIV prevention. Effects of the ring on sex for participants and their partners were explored at product use end visit (PUEV) in the subset who reported having sex during the study. Self‐reported adherence and acceptability of TFV versus placebo ring were compared using Fisher's exact test and Mann−Whitney U test, respectively. Exact binomial 95% confidence intervals for estimated proportions were calculated using the Pearson−Clopper method. Mean and SD of residual TFV levels in used rings were determined. In post‐hoc analysis, differences between low and high residual TFV concentrations were assessed using Fisher's exact test or logistic regression for categorical and Mann−Whitney U test for continuous participant characteristics. Differences in Nugent scores and having detectable concentrations of microbiota in participants with low versus high residual TFV concentrations across time points were assessed using generalized estimating equations (GEE) with repeated measures, binomial distribution and log link. Due to small sample sizes in subgroups and multiple comparisons across microbiota, differences were assessed across all time points. Nugent scores of 0−3, reflective of Lactobacillus predominant flora, were compared to scores greater than 3. All analyses were generated using SAS® (version 9.4) and R software (version 4.1.3).

## 3 RESULTS

**Figure 2:**
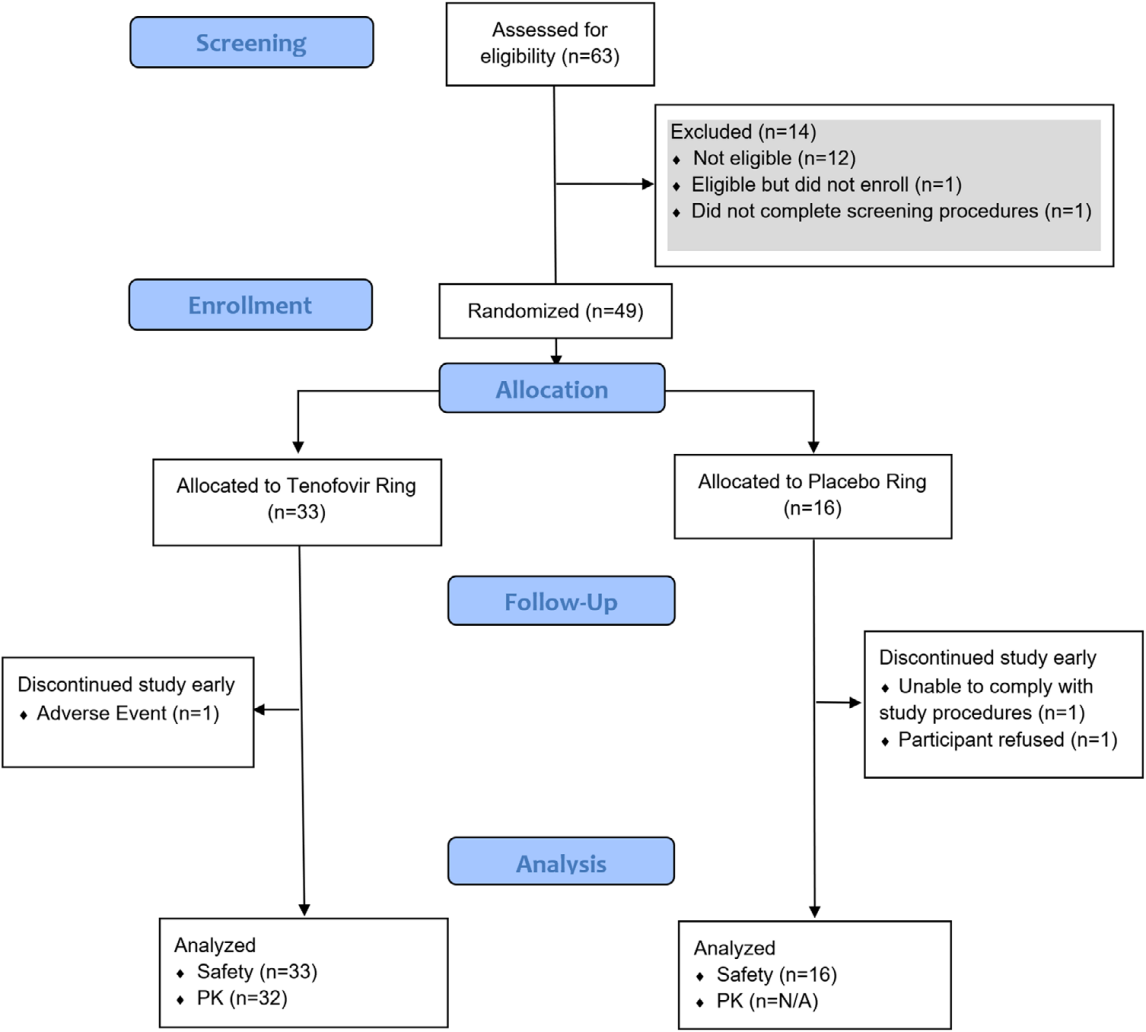
Flowchart of study participants.

From January to June 2019, 63 participants were screened and 49 enrolled (21 in Pittsburgh, 15 in San Francisco and 13 in Birmingham; see Figure 2). Participant demographics are outlined in Table 1. Mean age was 29.5 (range 18−41). Three participants did not complete the study: one participant (TFV) discontinued the study after the day 7 visit due to grade 1 AEs related to study product (vaginal erythema, uterine spasm and metrorrhagia); one participant (placebo) discontinued study product after the day 56 visit due to not wanting a foreign body inserted, after developing pelvic inflammatory disease and missed their final visit; the third participant (placebo) was discontinued after the day 56 visit due to missing subsequent visits (no reason available). Two participants refused all sample collection at the day 56 or 91 visit.

**Table 1:** Demographics and study‐related characteristics of participants in MTN‐038 by study arm

		Tenofovir ring	Placebo ring	Total
*N*		33	16	49
Age, years	Mean (SD)	29.5 (6.8)	29.1 (6.9)	29.4 (6.7)
	Range	18, 41	20, 43	18, 43
Race	Asian	4 (12%)	1 (6%)	5 (10%)
	Black	8 (24%)	3 (19%)	11 (22%)
	White	18 (55%)	8 (50%)	26 (53%)
	Mixed race or other	3 (9%)	4 (25%)	7 (14%)
Ethnicity	Latina/Hispanic	2 (6%)	1 (6%)	3 (6%)
Gender identity^a^	Female	32 (97%)	14 (88%)	46 (94%)
	Gender non‐conforming/gender variant	1 (3%)	2 (13%)	3 (6%)
Type of sex partner(s)	Male and female partners	4 (12%)	3 (19%)	7 (14%)
	Exclusively female partners	3 (9%)	2 (13%)	5 (10%)
	Exclusively male partners	19 (58%)	9 (56%)	28 (57%)
	NA (no sex partners)	7 (21%)	2 (13%)	9 (18%)
Number of participants with completed study visits, by visit	Day 7 visit	33 (100%)	16 (100%)	49 (100%)
	Day 14, 26 and 56 visits	32 (97%)	16 (100%)	48 (98%)
	Day 91 visit/PUEV^b^	33 (100%)	15 (94%)	48 (98%)
	Final contact visit	32 (97%)	14 (88%)	46 (94%)
# reporting sex during the study (*N* = 48^c^)	Yes	22 (67%)	12 (80%)	34 (71%)

Abbreviations: *N*, number; NA, not applicable; PUEV, product use end visit.

^a^ Response options for gender identity included: male, female, transgender male, transgender female, gender non‐conforming/gender variant, self‐identify.

^b^ One participant discontinued the study early after day 7 and that participant's last visit (on study day 15) is reflected in the day 91/PUEV summary.

^c^ One participant, assigned the placebo ring arm, did not complete the behavioural survey on day 91/PUEV.

### 3.1 Safety assessment

Overall, 113 AEs were reported in 78% (38/49) of participants (TFV 71 AEs among 76% [25/33], placebo 42 AEs among 81% [13/16]). Overall, 40% (45/113) of AEs reported in 26 participants were assessed as related to the study product. The most common related AEs included vaginal discharge (18 participants), vulvovaginal candidiasis (4), vaginal odour (3), uterine spasm (3) and dysmenorrhea (2). All AEs were grade 1 (80% [90/113]) or grade 2 (20% [23/113]). We found no statistically significant difference in the proportion of participants with grade ≥2 genitourinary AEs in TFV arm (12% [4/33] compared with placebo (25% [4/16], *p* = 0.41). There were no grade > = 3 AEs in either arm. AE rates did not increase with time on the study product or increased drug levels. While one participant in the TFV arm had vulvar ulceration due to excoriation from yeast infection, no participants in either arm had vaginal epithelial disruptions. No social harms were noted.

### 3.2 Pharmacokinetic analysis

**Figure 3:**
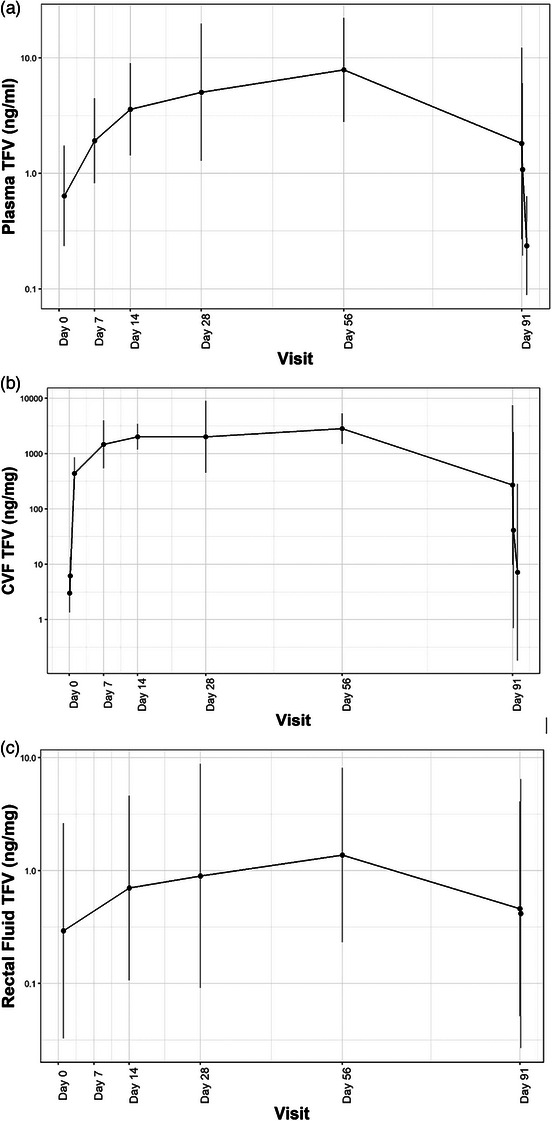
Geometric means of tenofovir (TFV) concentration in (a) blood plasma (ng/ml) and (b) cervico‐vaginal fluid (ng/mg), and (c) rectal fluid (ng/mg). Vertical bars indicate the exponentiated mean +/− the standard deviation of the log values.

Concentration‐time curves and geometric mean TFV concentrations in plasma, CVF, RF and cervical tissue are summarized in Figure 3 and Table 2, respectively, with median and interquartile

**Table 2:** Geometric mean tenofovir concentrations and PK parameters in plasma, cervicovaginal fluid, rectal fluid and cervical tissue

	TFV in plasma (CV%) (ng/ml), #BLQ^a^	TFV in CVF (CV%) (ng/mg), #BLQ^a^	TFV in rectal fluid (CV%) (ng/mg), #BLQ^a^	TFV in cervical tissue (CV%) (ng/mg)^b^, #BLQ^a^	TFV‐DP in cervical tissue (CV%) (fmol/mg)^b^, #BLQ^a^
Day 1	0.64 (131%), 6	438 (75%), 0	0.29 (1111%), 6	–	–
Day 7	1.92 (102%), 0	1468 (130%), 0	–	–	–
Day 14	3.59 (115%), 0	2006 (57%), 0	0.70 (583%), 2	45.9 (11,800%), 1	2680 (83%), 0
Day 28	5.04 (235%), 1	2012 (289%), 0	0.90 (1356%), 3	39.1 (323%), 0	3218 (86%), 0
Day 56	7.86 (140%), 0	2817 (71%), 0	1.38 (476%), 0	37.1 (177%), 0	1893 (178%), 0
Day 91, prior to removal	1.82 (612%), 8	271 (24,859%), 0	0.46 (1093%), 8	6.5 (25,4896%), 2	456 (16,736%), 0
Day 91, 4 hours post removal	1.08 (427%), 10	41.3 (40,6784%), 2	0.42 (4316%), 7	–	–
Day 92, 24 hours post removal	0.24 (127%), 20	7.2 (85,571%), 2	–	–	–
C_max_	11.98 (121%)	3853 (59%)	5.88 (1064%)	–	–
T_max_ (days)	59 (54%)	33.5 (106%)	33.6 (157%)	–	–
AUC (0−92)^c^	599 (99.9%)	216 (49%)	167 (404%)	–	–

Abbreviations: AUC, area under the concentration time curve from 0 to 92 days; C_max_, peak concentration; CVF, cervicovaginal fluid; CV, geometric coefficient of variation; fmol/mg, femtomole per milligram; ng/mg, nanogram per milligram; ng/ml, nanogram per millilitre; PK, pharmacokinetic; TFV, tenofovir; T_max_, time to peak concentration.

^a^ Number of participants with concentrations below the lower limit of quantitation.

^b^ *n* = 15 for day 14 and day 56 biopsies; *n* = 17 for day 28 and day 91 biopsies.

^c^ AUC in ng/ml × days for plasma and ng/mg × days for CVF and rectal fluid.

concentrations are shown in Table S1. In plasma, geometric mean TFV concentrations were low at day 1 (0.64 ng/ml) and increased through day 56 (7.86 ng/ml), with T_max_ = 59.0 days and C_max_ = 11.98 ng/ml. Geometric mean plasma TFV concentrations subsequently declined to 1.81 ng/ml at day 91 prior to ring removal, and further declined to 0.24 ng/ml 24 hours post‐removal (below limit of quantification). Inter‐individual variability was high across time points (geometric coefficient of variation [CV] 102−612%) and highest at day 91 prior to ring removal.

In CVF, geometric mean TFV concentrations were quantifiable 1 and 4 hours post‐insertion (3.0 and 6.2 ng/mg), and rapidly increased to 438 ng/mg at day 1, with a T_max_ = 33.5 days and C_max_ = 3853 ng/mg. Geometric mean CVF TFV concentrations substantially declined to 271 ng/mg at day 91 prior to ring removal, and further decreased to 7.2 ng/mg 24 hours post‐removal. Inter‐individual variability was moderate to high across time points (CV 57−406,784%) and was highest at day 91 pre‐ and post‐removal.

Geometric mean RF TFV concentrations slowly increased over time but remained relatively low, with a T_max_ = 33.6 days and C_max_ = 5.88 ng/ml. Geometric mean RF TFV concentrations decreased to 0.46 ng/mg at day 91 prior to removal and were relatively similar 4 hours post‐removal (0.42 ng/mg). Inter‐individual variability was high across time points (CV 476−4316%), highest at day 91 4‐hours post‐removal.

In cervical tissue, geometric mean TFV concentrations were highest at day 14 (45.9 ng/mg), declined slightly at days 28 (39.1 ng/mg) and 56 (37.1 ng/mg), and were substantially lower at day 91 prior to ring removal (6.5 ng/mg). Geometric TFV‐DP concentrations were highest at day 28 (3218 fmol/mg), and then steadily declined through days 56 (1893 fmol/mg) and 91 (456 fmol/mg). The inter‐individual variability for both TFV and TFV‐DP in cervical tissue was highest at day 91.

### 3.3 Residual TFV in collected rings

**Figure 4:**
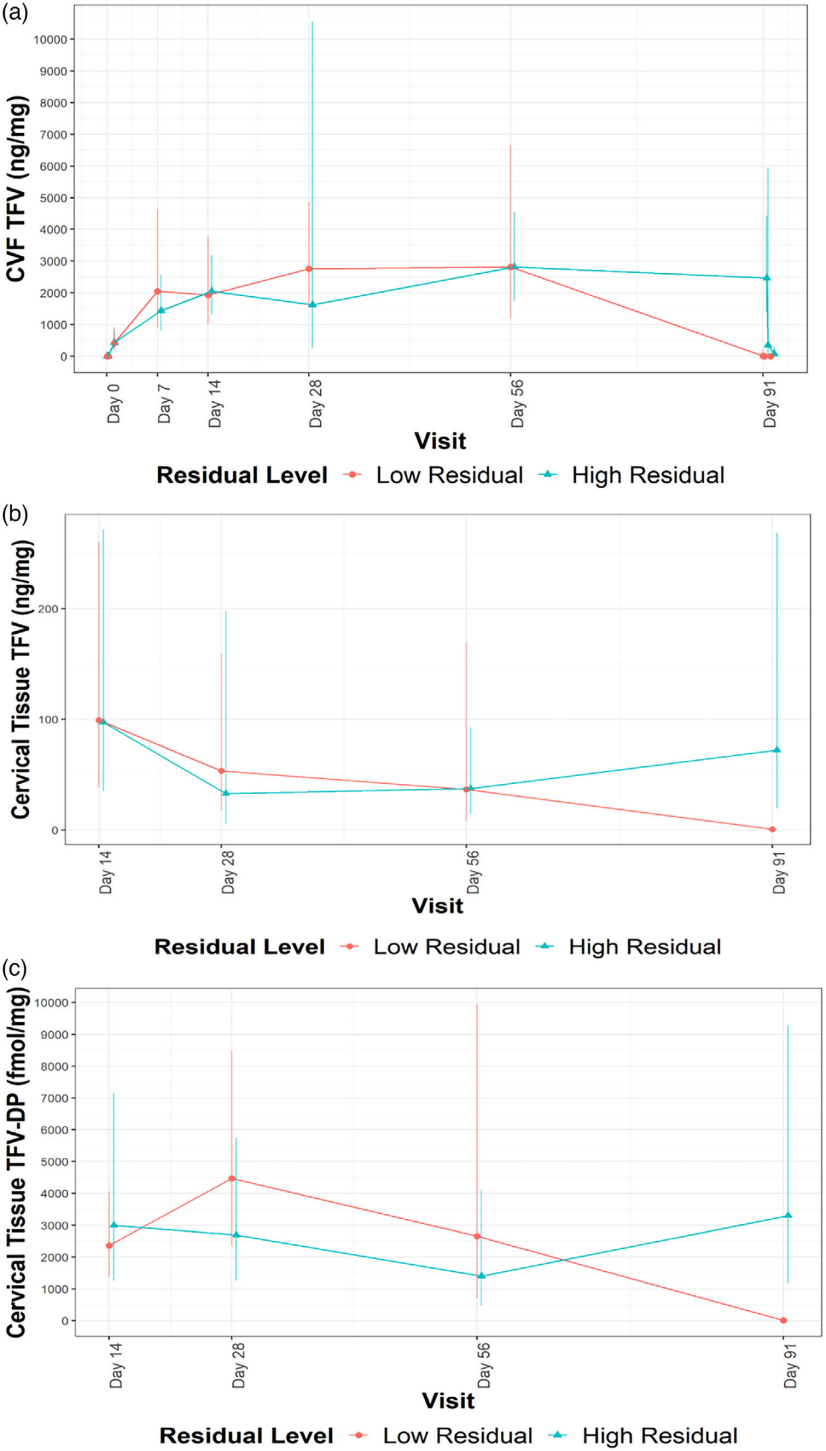
Geometric mean drug concentrations by low versus high residual TFV in returned rings for (a) TFV in cervicovaginal fluid (CVF) (ng/mg), (b) TFV in cervical tissue (ng/mg) and (c) TFV‐DP in cervical tissue (fmol/mg).

At day 91, mean and median residual TFV levels in used rings with the active drug were 0.29 and 0.15 g, respectively (IQR: 0.00−0.58 g). One ring was not returned from a participant who discontinued the study early. Among 32 returned TFV rings, 13 had 0 or trace (<0.1 mg) residual TFV. Participants who had no or trace residual TFV in collected rings had somewhat higher TFV and TFV‐DP concentrations in CVF and cervical tissue through day 56 but substantially lower levels at day 91, while those with high residual TFV had high CVF and tissue concentrations through day 91, likely contributing to the high day 91 inter‐individual variability (Figure 4). In post‐hoc analysis, residual TFV concentrations in returned rings did not differ significantly by baseline socio‐demographics, ring outages or sexual activity (Table S2). There was no difference in Nugent score between low and high residual groups (GEE *p* = 0.14). While detection and median log_10_ concentrations of anaerobic bacteria (*Gardnerella*, *Prevotella* and *Atopobium* species) were generally higher in participants with low versus high residual TFV concentrations, these differences were not statistically significant (Table S3).

### 3.4 Adherence and acceptability

Most participants reported being fully adherent during the study: TFV 85% (95% CI 68−95%), placebo 81% (95% CI 54−96%) with no statistically significant differences between groups (*p* = 1.00). Among eight participants who were not fully adherent, three terminated early, two had a product hold, two reported a single outage, one reported two outages and four reported three outages. The median duration of ring outage was 13.7 hours, with three (6%) participants with total outages of less than 30 minutes. Reported reasons for ring removal included discomfort or other symptoms (2 participants), ring partially falling out (2), to clean the ring (2), ring placement or checking presence (2) and menses/bleeding (1). Seven ring expulsions were reported among three participants: three related to sex, and four related to menses.

**Figure 5:**
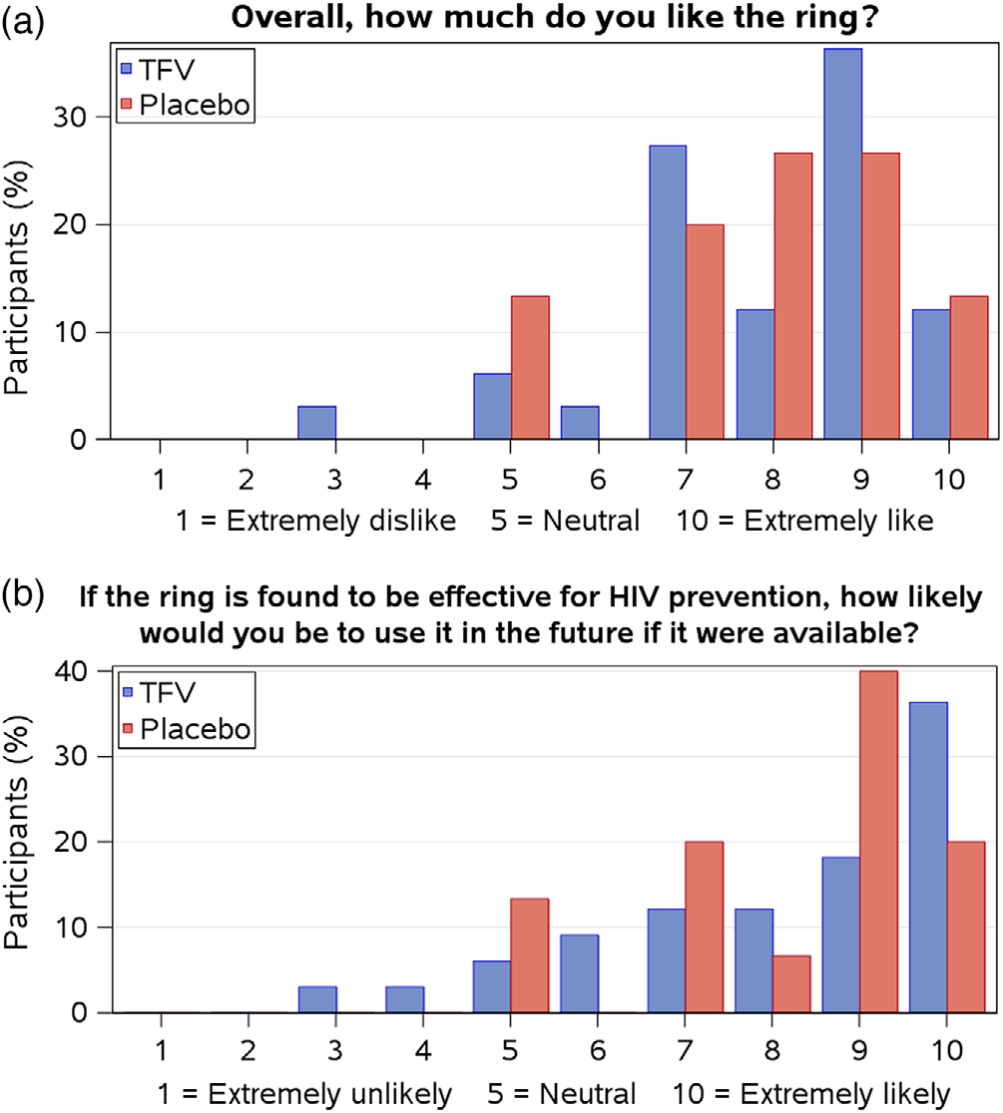
Acceptability (a) and future willingness to use (b) the tenofovir ring.

Acceptability of the ring at the study exit was high (Figure 5). Median acceptability scores were 8/10 (IQR 7−9) in both arms, with no difference between arms (*p* = 0.94). Overall, 84% (95% CI 70−93%) of participants gave the vaginal ring an acceptability score as high or higher than they gave male condoms (90% TFV, 71% placebo). Most participants (56%) reported a high score (9 or 10) when asked how likely they would be to use the vaginal ring if effective (median score: 9, IQR 7−10). When asked about their ring experiences during sex (Table 3), among 34 women who had sex during the study, a majority reported they never felt it (53%) and half of their partners never felt it or it did not bother them (50%).

**Table 3:** Overall experiences of the ring during sex among sexually active participants in MTN‐038 at PUEV/91‐day visit

Ring and sex questions	Responses^a^	TOTAL % (*N* = 34)
Did ppt mind wearing ring during sex? (J16)	YES	2 (6%)
	NO	32 (94%)
Ever felt the ring inside and botherness level when had sex? (J12+J13)	Never felt ring	18 (53%)
	Felt ring/not bothered	6 (18%)
	Felt ring/bothered^b^	10 (29%)
Did ring affect participant's sexual pleasure (J17)	Increased pleasure	3 (9%)
	Decreased pleasure	2 (6%)
	No change	29 (85%)
Partner ever felt the ring during sex and botherness level? (J14+J15)	Never felt ring	5 (15%)
	Felt ring/not bothered	12 (35%)
	Felt ring/bothered^c^	16 (47%)
	DK	1 (3%)
Did ring affect partner's sexual pleasure (J18)	Increased pleasure	0 (0%)
	Decreased pleasure	5 (15%)
	No change	23 (68%)
	DK	6 (18%)
Did any partner ever ask participant to stop using the ring? (J20)	YES	0 (0%)
	NO	34 (100%)

Abbreviation: DK, don't know.

^a^ One participant who did not complete the final questionnaire and 14 participants who reported not being sexually active are excluded.

^b^ Responded a little (8), somewhat (1) or very much (1) bothered.

^c^ Responded a little (10), somewhat (4) or very much (2) bothered.

## 4 DISCUSSION

In this phase 1 study, the TFV ring was safe, well‐tolerated, and acceptable overall and during sex when used continuously over 90 days. All AEs were mild or moderate in severity, and the majority were unrelated to the study product. While the most commonly reported AEs were genitourinary in nature, there were no significant differences in the proportion of participants with grade 2 or higher genitourinary AEs across arms. An earlier phase 1 study of a different vaginal ring releasing TDF was stopped early due to genital ulcerations observed in 8/12 women assigned to the TDF ring, with ulcers detected 23−56 days after ring insertion [29]. These findings were not observed in CONRAD 138, a 90‐day phase 1 study of TFV and levonorgestrel (LNG) vaginal ring in sexually active women, in which only one of 36 women receiving TFV/LNG ring presented with a 1 cm epithelial disruption which resolved within 10 days of ring removal [30]. With no vaginal ulcerations observed in either arm, our study provides additional safety data that the TFV ring does not result in genital ulceration when used by healthy sexually active women, in contrast to the TDF ring assessed previously.

While plasma TFV concentrations were low across all time points, as expected with topical dosing of TFV, we observed high geometric mean TFV concentrations in CVF achieved by day 7 (1468 ng/mg) and increasing through day 56 (2817 ng/mg). In CAPRISA‐004, a CV aspirate TFV concentration of >1000 ng/ml was associated with a 76% protection against HIV‐1 [31, 32]. Based on PK and efficacy studies of TFV gel in macaques, a benchmark of 1000 fmol/mg for TFV‐DP was established as highly protective in simian human immunodeficiency virus challenge studies [33, 34]. In MTN‐038, this target was exceeded in biopsy samples at days 14, 28 and 56, with TFV‐DP concentrations ranging from 1893 to 3218 fmol/mg. Similarly, high cervical tissue TFV concentrations (37.1−45.9 ng/mg) were achieved through day 56, above the >10 ng/mg benchmark associated with high TFV‐DP tissue concentrations as used in CONRAD 138 [30]. RF concentrations were low, consistent with prior studies of vaginal and rectal administration of TFV gel in macaques and in CONRAD 138 [30, 35], and likely non‐protective.

Across all compartments, we observed marked declines in TFV and TFV‐DP concentrations at day 91. In an analysis of returned rings, 13 of 32 participants in the TFV group had undetectable or low residual TFV. In post‐hoc analysis, participants with higher residual TFV in returned rings had high TFV and TFV‐DP concentrations in CVF and cervical tissue at day 91, whereas those with no/trace residual TFV in returned rings had somewhat higher TFV and TFV‐DP concentrations at early time points and significantly lower concentrations at day 91 (Figure 3a−c). These findings suggest that TFV release from the ring was higher in a substantial minority (∼40%) of participants, resulting in depletion of drug from the ring by day 91. Additionally, we cannot rule out the possibility that lower TFV concentrations in blood and tissue after day 56 were due to more frequent, unreported ring removals.

In CONRAD 138, 17 of 37 (46%) women receiving the TFV/LNG ring had high *in vivo* release of TFV as defined as ≥98% drug release from the ring [36]. This higher release rate was observed in participants with a diverse set of anaerobic vaginal microbiota (community state type [CST] IV A/B) during follow‐up and was associated with lower TFV‐DP in vaginal tissue at the end of treatment, compared with participants with Lactobacillus‐dominated microbiota. Despite these findings, HIV‐1 inhibitory activity *in vitro* was significantly increased from baseline in all participants regardless of vaginal microbiota. It has been hypothesized that higher vaginal pH associated with anaerobic microbiota may result in increased TFV release rates due to increased solubility of TFV at higher pH [15]. Additionally, prior studies have observed the uptake and degradation of TFV by CST‐IV vaginal microbiota [37, 38].

In MTN‐038, residual TFV concentrations in returned rings did not differ by baseline characteristics, and there were no statistically significant differences in the detection of 11 microbiota measured in this study between participants with no/low versus high residual TFV. Differences observed across these studies regarding the association of vaginal microbiota and TFV ring release may be due to different methods used in analysing microbiota data (individual microbiota in MTN‐038 vs. CST in CONRAD 138, as multiple anaerobic bacteria collectively may contribute to increased release rates). Additional studies are needed to better understand the mechanisms for rapid TFV release and individuals who are more likely to have this occur. Regardless of the mechanism, higher TFV ring loading may be required to ensure adequate TFV drug release over a 90‐day period for the entire population of users. Additionally, improvements in ring technology to ensure a more consistent release rate across conditions can be pursued.

This study has several limitations. First, vaginal pH was not measured and we, therefore, were unable to evaluate whether differences in residual TFV levels were related to vaginal pH. Additionally, analysis of microbiota by low versus high residual TFV was done post‐hoc, and due to small sample sizes in these subgroups and multiple comparisons across microbiota, we were only able to assess differences across all time points. Also, social desirability may have biased self‐reported adherence and acceptability. Furthermore, because eligibility did not require sexual activity, we asked limited questions on ring use during sex. Nevertheless, for a majority, during sex, this ring was not felt by themselves nor their partners, or if felt, was not bothersome. This aligns with qualitative findings from this study [39] and the ring acceptability literature [40, 41]. This study had several strengths, including enrolling a diverse cohort across three geographically distinct US sites, having a placebo control arm, and including PK and safety measures at multiple time points over 91 days of follow‐up.

## 5 CONCLUSIONS

In summary, we found the 90‐day TFV ring to be safe and well‐tolerated, with high acceptability in a diverse population. Target cervical tissue concentrations of TFV and TFV‐DP were exceeded through day 56, but declined by day 91 in a subset of participants due to greater TFV release from the rings. Additional studies should investigate the aetiology of differential TFV ring release rates, and vaginal rings with higher TFV loading should be evaluated for PK and safety to optimize the duration of use and HIV protection.

## COMPETING INTERESTS

AYL has received funding for investigator‐sponsored research grants from Gilead Sciences and Viiv Healthcare, and has led studies in which Gilead Sciences has donated study drugs. MAM has received research support from Viiv Healthcare, Gilead Sciences, Merck Inc. and Ridgeback Therapeutics. HG, BR, BAC, CH, AVDS, AB, MB, JR, CEJ, RS, KB, JS, AT, JMP and MAM declare that they have no competing interests.

## AUTHORS’ CONTRIBUTIONS

AYL, HG, BR, BAC, CH, AVDS, AB, MB, JR, CEJ, RS, KB, JMP and MAM assisted with protocol development and design of the analysis. AYL, BAC and CH collected the data. HG, BR, MB and MAM conducted the analysis. AYL and HG wrote the manuscript. All authors reviewed, edited and approved the final manuscript.

## FUNDING

The study was designed and implemented by the Microbicide Trials Network (MTN). The MTN was funded by the National Institute of Allergy and Infectious Diseases (UM1AI068633, UM1AI068615, UM1AI106707), with co‐funding from the Eunice Kennedy Shriver National Institute of Child Health and Human Development and the National Institute of Mental Health, all components of the US National Institutes of Health. CONRAD developed the tenofovir ring and provided clinical supplies with funding from PEPFAR through a cooperative agreement between the US Agency for International Development (USAID) and Eastern Virginia Medical School (AID‐OAA‐A‐14‐00010).

## DISCLAIMER

The content is solely the responsibility of the authors and does not necessarily represent the official views of the National Institutes of Health.

## Supporting information


**Table S1**: Median (IQR) Tenofovir Concentrations and PK Parameters in Plasma, Cervicovaginal Fluid, Rectal Fluid, and Cervical Tissue
**Table S2**: Demographics and baseline characteristics, by low vs. high residual TFV in returned rings
**Table S3**: Vaginal Microbiota detection and median log_10_ concentrations, by low vs. high residual TFV in returned rings

## Data Availability

The data that support the findings of this study are available from the corresponding author upon reasonable request.
