# Supplementary material for: Phase 1 randomized pharmacokinetic and safety study of a 90‐day tenofovir vaginal ring in the United States
Source: J Int AIDS Soc. 2024 Mar 5;27(3):e26223. doi: 10.1002/jia2.26223 (PMC10935712; doi:10.1002/jia2.26223)
Supplement: Supplementary file 1 — Table S1: Median (IQR) Tenofovir Concentrations and PK Parameters in Plasma, Cervicovaginal Fluid, Rectal Fluid, and Cervical Tissue Table S2: Demographics and baseline characteristics, by low vs. high residual TFV in returned rings Table S3: Vaginal Microbiota detection and median log10 concentrations, by low vs. high residual TFV in returned rings [file JIA2-27-e26223-s001.docx]

Supplementary Table 1: Median (IQR) Tenofovir Concentrations and PK Parameters in Plasma, Cervicovaginal Fluid, Rectal Fluid, and Cervical Tissue

|  | **TFV in Plasma**  **(ng/mL)** | **TFV in CVF**  **(ng/mg)** | **TFV in Rectal Fluid (ng/mg)** | **TFV in Cervical Tissue (ng/mg)^1^** | **TFV-DP in Cervical Tissue (CV%) (fmol/mg) ^1^** |
| --- | --- | --- | --- | --- | --- |
| Day 1 | 0.69 (0.45, 1.31) | 529 (387, 702) | 0.32 (0.10, 0.69) | -- | -- |
| Day 7 | 1.84 (0.98, 3.49) | 1707 (873, 2389) | -- | -- | -- |
| Day 14 | 3.41 (1.88, 6.83) | 2108 (1431, 2829) | 0.62 (0.21, 2.06) | 122 (38.5, 196) | 2752 (1812, 3927) |
| Day 28 | 5.95 (2.00, 12.95) | 2453 (2014, 3601) | 0.94 (0.26, 3.46) | 28.5 (13.7, 123) | 3454 (1593, 5980) |
| Day 56 | 8.06 (2.90, 16.50) | 3033 (1959, 4293) | 1.28 (0.47, 2.76) | 37.6 (27.7, 68.3) | 2125 (1217, 4043) |
| Day 91, prior to removal | 2.65 (0.17, 9.55) | 1300 (30.6, 3098) | 0.92 (0.06, 1.96) | 27.3 (0.7, 125) | 958 (135, 5168) |
| Day 91, 4 h post removal | 1.80 (BLQ, 4.81) | 135.5 (2.2, 1324) | 0.43 (0.06, 1.77) | -- | -- |
| Day 92, 24 h post removal | BLQ (BLQ, 0.53) | 24.3 (0.4, 146.5) | -- | -- | -- |
| C_max_ | 11.40 (6.37, 29.10) | 3514 (2612, 5193) | 3.12 (1.42, 13.95) | -- | -- |
| T_max_ (days) | 57.55 (55.94, 90.91) | 54.6 (15.1, 57.1) | 55.9 (28.0, 58.4) | -- | -- |
| AUC (0-92)^2^ | 621  (269, 1232) | 205,493  (158,719, 333,151) | 105,407  (43,640,417,667) | -- | -- |

Abbreviations: PK, pharmacokinetic; TFV, tenofovir; CVF, cervicovaginal fluid; ng/mL, nanogram per mililiter; ng/mg, nanogram per mililiter; fmol/mg, femtomole per milligram; h, hour; C_max_, peak concentration; T_max_, time to peak concentration; AUC, area under the concentration time curve from 0-92 days

^1^n=15 for day 14 and day 56 biopsies; n=17 for day 28 and day 91 biopsies

^2^AUC in ng/ml*days for plasma and ng/mg*days for CVF and rectal fluid

Supplementary Table 2: Demographics and baseline characteristics, by low vs. high residual TFV in returned rings

| Characteristic | Low residual TFV | High residual TFV | P value |
| --- | --- | --- | --- |
| Sites  Birmingham, AL  Pittsburgh, PA  San Francisco, CA | 3 (23%)  5 (38%)  5 (38%) | 6 (32%)  8 (42%)  5 (26%) | 0.75 |
| Age  Mean (SD) | 30.2 (6.9) | 29.3 (6.9) | 0.75 |
| Sexual orientation  Lesbian  Heterosexual  Bisexual  Queer | 2 (15%)  7 (54%)  2 (15%)  2 (15%) | 0 (0%)  13 (68%)  4 (21%)  2 (11%) | 0.47 |
| Gender identity*  Female  Gender nonconforming/gender variant | 12 (92%)  1 (8%) | 19 (100%)  0 (0%) | 0.41 |
| Biological gender of partners  Both male and female  Exclusively female partners  Exclusively male partners  No sex partners | 2 (15%)  1 (8%)  7 (54%)  3 (23%) | 2 (11%)  2 (11%)  12 (63%)  3 (16%) | 0.72 |
| Ethnicity  Hispanic/Latinx  Not Hispanic/Latinx | 2 (15%)  11 (85%) | 0 (0%)  19 (100%) | 0.16 |
| Race  Asian  Black  White  Other/Mixed | 2 (15%)  2 (15%)  8 (62%)  1 (8%) | 2 (11%)  5 (26%)  10 (53%)  2 (11%) | 0.72 |
| Level of education  Less than high school  Graduated from high school  Some college  College graduate or more | 1 (8%)  0 (0%)  3 (23%)  9 (69%) | 0 (0%)  1 (5%)  7 (37%)  11 (58%) | 0.71 |
| Housing status  Own a house or apartment  Rent a house or apartment  Rent a room in a group house  Stay with partners/relatives/family  Stay with friends | 2 (15%)  7 (54%)  2 (15%)  2 (15%)  0 (0%) | 5 (26%)  11 (58%)  0 (0%)  2 (11%)  1 (5%) | 0.67 |
| Ring outages  Fully adherent participants  Non-adherent participants | 13 (100%)  0 (0%) | 15 (79%)  4 (21%) | 0.13 |
| Nugent score  0-3  4-6  7-10  Missing | 7 (54%)  1 (8%)  4 (31%)  1 (8%) | 13 (68%)  1 (5%)  3 (16%)  2 (11%) | 0.58 |
| Penile vaginal sex in past 4 weeks (baseline)  No  Yes | 6 (46%)  7 (54%) | 11 (58%)  8 (42%) | 0.72 |
| Penile vaginal sex in past 4 weeks (follow-up)  No  Yes | 6 (46%)  7 (54%) | 8 (42%)  11 (58%) | 1.0 |

*Response options for gender identity included: male, female, transgender male, transgender female, gender nonconforming/gender variant, self-identify

Supplementary Table 3: Vaginal Microbiota detection and median log_10_ concentrations, by low vs. high residual TFV in returned rings

| Characteristic | Low residual TFV (N=13) | | High residual TFV (N=19) | | P value* |
| --- | --- | --- | --- | --- | --- |
|  | % Detected | Median (IQR) | % Detected | Median (IQR) |  |
| *Lactobacillus crispatus*  Enrollment  Day 28  Day 56  Day 91/PUEV | 46%  46%  38%  46% | BLQ (BLQ, 7.4)  BLQ (BLQ, 6.3)  BLQ (BLQ, 5.2)  BLQ (BLQ 6.6) | 63%  63%  63%  63% | 6.5 (BLQ, 7.6)  5.9 (BLQ, 7.4)  5.9 (BLQ, 6.6)  6.1 (BLQ, 6.9) | 0.29 |
| *Atopobium vaginae*  Enrollment  Day 28  Day 56  Day 91/PUEV | 38%  62%  62%  62% | BLQ (BLQ, 0.7)  5.5 (BLQ, 6.5)  3.8 (BLQ, 6.5)  4.8 (BLQ, 7.2) | 26%  37%  32%  32% | BLQ (BLQ, 5.8)  BLQ (BLQ, 5.6)  BLQ (BLQ, 3.9)  BLQ (BLQ, 4.3) | 0.21 |
| *Lactobacillus iners*  Enrollment  Day 28  Day 56  Day 91/PUEV | 85%  77%  85%  85% | 6.3 (5.1, 7.2)  5.7 (0.7, 6.8)  6.4 (6.2, 7.3)  6.9 (4.4, 7.4) | 63%  63% 74%  74% | 5.9 (BLQ, 7.5)  5.2 (BLQ, 7.2)  6.5 (BLQ, 7.3)  5.5 (BLQ, 7.4) | 0.25 |
| *Megaspheaera lornae*  Enrollment  Day 28  Day 56  Day 91/PUEV | 31%  23%  31%  31% | BLQ (BLQ, 5.5) BLQ (BLQ, BLQ)  BLQ (BLQ, 6.5)  BLQ (BLQ, 5.3) | 21%  21%  16% 11% | BLQ (BLQ, BLQ) BLQ (BLQ, BLQ)  BLQ (BLQ, BLQ)  BLQ (BLQ, BLQ) | 0.44 |
| *Gardnerella vaginae*  Enrollment  Day 28  Day 56  Day 91/PUEV | 69%  77%  85%  85% | 3.8 (BLQ, 6.5)  7.1 (4.3, 7.4)  6.9 (0.7, 7.7)  6.3 (4.5, 7.9) | 58%  63%  58%  63% | 0.8 (BLQ, 5.2)  3.4 (BLQ, 6.4)  4.2 (BLQ, 6.9)  3.5 (BLQ, 6.6) | 0.28 |
| *Prevotella bivia*  Enrollment  Day 28  Day 56  Day 91/PUEV | 31%  77%  77%  54% | BLQ (BLQ, 3.5)  3.8 (2.9, 4.5)  3.3 (2.7, 4.8)  0.6 (BLQ, 4.2) | 21%  32%  37% 26% | BLQ (BLQ, BLQ))  BLQ (BLQ, 3.2)  BLQ (BLQ, 3.2)  BLQ (BLQ, 3.1) | 0.11 |
| *Sneathia/Leptotrichia* species  Enrollment  Day 28  Day 56  Day 91/PUEV | 31%  23%  31%  38% | BLQ (BLQ, 5.3)  BLQ (BLQ, BLQ)  BLQ (BLQ, 6.2)  BLQ (BLQ, 6.5) | 21%  21%  16% 21% | BLQ (BLQ, BLQ)  BLQ (BLQ, BLQ)  BLQ (BLQ, BLQ)  BLQ (BLQ, BLQ) | 0.45 |
| *Eggerthella*-like species  Enrollment  Day 28  Day 56  Day 91/PUEV | 31%  23%  38%  38% | BLQ (BLQ, 0.7)  BLQ (BLQ, BLQ)  BLQ (BLQ, 6.1)  BLQ (BLQ, 6.5) | 21% 21%  16%  21% | BLQ (BLQ, BLQ)  BLQ (BLQ, BLQ)  BLQ (BLQ, BLQ)  BLQ (BLQ, BLQ) | 0.40 |
| *Prevotella amnii*  Enrollment  Day 28  Day 56  Day 91/PUEV | 15% 15%  15%  31% | BLQ (BLQ, BLQ)  BLQ (BLQ, BLQ)  BLQ (BLQ, BLQ)  BLQ (BLQ, 4.2) | 21%  21%  16% 16% | BLQ (BLQ, BLQ)  BLQ (BLQ, BLQ)  BLQ (BLQ, BLQ)  BLQ (BLQ, BLQ) | 0.91 |
| *Prevotella timonensis*  Enrollment  Day 28  Day 56  Day 91/PUEV | 62%  85%  77%  69% | 4.7 (BLQ, 5.6)  4.2 (3.3, 5.5)  4.3 (0.4, 6.7)  5.6 (BLQ, 6.8) | 58%  58%  47%  47% | 0.5 (BLQ, 5.2)  0.6 (BLQ, 5.2)  BLQ (BLQ, 4.4)  BLQ (BLQ, 5.5) | 0.25 |
| *Mycoplasma hominis*  Enrollment  Day 28  Day 56  Day 91/PUEV | 7%  0%  7%  23% | BLQ (BLQ, BLQ)  BLQ (BLQ, BLQ)  BLQ (BLQ, BLQ)  BLQ (BLQ, BLQ) | 11%  16%  21%  5% | BLQ (BLQ, BLQ)  BLQ (BLQ, BLQ)  BLQ (BLQ, BLQ)  BLQ (BLQ, BLQ) | 0.65 |

BLQ = below the limit of quantitation
*Unadjusted P values for generalized estimating equations (GEE) results for low vs. high residual drug levels for binomial outcome (BLQ vs. above) for each microbiota type.
